# Cocoa bean metabolomics reveals polyphenols as potential markers relating to fine dark chocolate color shades

**DOI:** 10.3389/fnut.2024.1467282

**Published:** 2024-09-25

**Authors:** Aécio Luís de Sousa Dias, Emmanuelle Meudec, Arnaud Verbaere, Sophie Lair, Jean-Claude Boulet, Nicolas Sommerer

**Affiliations:** ^1^SPO, Université de Montpellier, INRAE, Institut Agro, Montpellier, France; ^2^INRAE, PROBE Research Infrastructure, PFP Polyphenol Analysis Facility, Montpellier, France; ^3^Valrhona, Tain-l'Hermitage, France

**Keywords:** *Theobroma cacao*, cocoa beans, chocolate, metabolomics, polyphenols, flavanols, procyanidins, discriminating compounds

## Abstract

**Introduction:**

This study aimed to evaluate the color and the discriminating compounds for two types of cocoa beans (black and brown beans) related to 70% dark chocolates of black and brown colors from a previous work of our group.

**Methods:**

Color analysis and untargeted high-resolution mass spectrometry-based metabolomic analysis were performed on eight beans of each type. Mass spectral data processing, univariate and multivariate statistical methods were conducted for classification of beans and selection of discriminant features.

**Results and discussion:**

The results showed that the color difference already observed for black and brown chocolates preexists in the beans. Black and brown beans had 45 and 50 discriminant features, respectively, of which 16 and 41 were phenolic compounds. Most of them were also previously identified as discriminating compounds for black and brown chocolates. Black beans predominantly contained glycosylated flavanols, ranging from monomers to trimers, with dimers and trimers being A-type procyanidins, along with a phenolic acid (protocatechuic acid), and an O-glycosylated flavonol (quercetin-3-O-glucoside). In contrast, brown beans mostly contained non-glycosylated B-type procyanidins ranging from dimers to decamers, but also dimers and trimers of A-type procyanidins, and a glycosylated and sulfated flavanol ((epi) catechin hexoside-sulfate). These markers may be useful for quality control purposes and may contribute to the selection of beans that yield black or brown dark chocolates.

## 1 Introduction

*Theobroma cacao* L. is a tree native to the Amazon region of South America (1) and is classified into 10 different genetic clusters (2). It is primarily cultivated in Africa, Latin America, and Asia for the production of beans used in chocolate manufacturing (3). The processing of cocoa beans into chocolates is a complex process, involving stages of fermentation and drying in their countries of origin, followed by roasting, grinding, conching, and tempering in the countries that produce and market the chocolates (4).

Chemical composition of cocoa beans depends on their genetic and geographical origins, agronomic practices, climatic, and processing conditions (5). Cocoa flavor chemistry involves non-volatile compounds such as phenolic compounds, alkaloids, proteins, (oligo)peptides, and carbohydrates, and volatile compounds, including alcohols, aldehydes, ketones, esters, pyrazines, and acids (6). Cocoa oligopeptides are degradation products formed through proteolytic cleavage of cocoa proteins during fermentation, primarily due to the action of endogenous enzymes (7).

Particularly, phenolic compounds in cocoa-derived products are an important class because they contribute to sensory characteristics such as color, astringency, and bitterness, and exhibit anticarcinogenic, anti-atherosclerotic, anti-inflammatory, and antioxidant properties (8). They can also serve as markers that can contribute to quality control, traceability, and authenticity of the products (9). The phenolic compounds in cocoa beans are primarily flavanols, but also include anthocyanins, flavones, flavonols, and phenolic acids (6, 10). The main flavanols found in beans are epicatechin, catechin, and their oligomers and polymers known as procyanidins (8), which can be categorized as B-type and A-type. B-type procyanidins have monomers linked through a C4–C8 or C4–C6 bond, whereas an additional C2–O–C7 or C2–O–C5 bond is present in A-type procyanidins (11). Overall, the total amount of phenolic compounds is significantly reduced during cocoa processing (5). The chemical transformations of flavanols during processing include epimerization (12, 13), oxidation (14, 15), polymerization, and depolymerization (16, 17).

Untargeted metabolomic studies on cocoa-derived products were able to identify chemical markers, including phenolic compounds, to distinguish samples according to the cultivar (18) and geographic origin (19), as well as related to fermentation (19, 20), drying, roasting processes (21) and chocolate types (22, 23). Additionally, feature-based molecular networking (FBMN) analysis, which organizes tandem mass spectrometry (MS/MS) data set according to the MS/MS spectral similarity, has been included in metabolomic workflows to improve the annotation process (22, 23). Indeed, molecular networks group compounds that often belong to the same chemical family, so that unknown compounds may be annotated based on previously annotated compounds when they are connected in a same cluster (24).

In particular, two types of high-quality dark chocolates with 70% cocoa content were studied due to their distinct colors (black and brown) (23). This study revealed that each type of chocolate had different kinds of discriminating phenolic compounds. In general, black chocolates contained glycosylated flavanols and A-type procyanidins having a degree of polymerization up to 3, while brown chocolates had predominantly non-glycosylated B-type procyanidins having higher degree of polymerization. As these types of chocolate were produced from commercial cocoa beans using the same process, these cocoa beans likely also have different chemical profiles and may be differentiated by chemical markers. However, no study has been conducted on either the chemical profiles or the colors of the raw materials of those chocolates.

Therefore, the aim of this study was to evaluate the color and the discriminating compounds for two types of cocoa beans sourced from the same batch of the raw materials used to produce the dark chocolates of black and brown colors studied in a previous work (23), employing an untargeted metabolomic approach based on high-resolution mass spectrometry (HRMS).

## 2 Materials and methods

### 2.1 Materials

Standards of procyanidin dimer A2 and protocatechuic acid were purchased from Sigma-Aldrich (Steinheim, Germany). Procyanidin B2 was obtained from Extrasynthese (Genay, France), procyanidin B5 from TransMIT PlantMetaChem (Giessen, Germany), and quercetin-3-*O*-glucoside from Phytolab (Vestenbergsgreuth, Germany). LC–MS grade acetonitrile and LC–MS grade formic acid were acquired from Biosolve (Valkenswaard, The Netherlands) and purified water was obtained from a Milli-Q water Millipore system (Bedford, MA, United States). Hydrochloric acid solution (6 M HCl) was purchased from Honeywell Fluka (Seelze, Germany).

### 2.2 Cocoa bean samples

In our previous work, eight black chocolates and eight brown chocolates were selected and studied (23). In the present work, the cocoa beans corresponded to the ones used for the production of those chocolates. By analogy with the previous work, the cocoa beans were named as black and brown beans. Black cocoa bean samples were coded as F178, F181, F182, F206, F214, F215, F216 and F217, whereas brown cocoa bean samples were named F15, F50, F51, F54, F57, F184, F185 and F186. They corresponded to raw commercial fermented and dried cocoa beans and were provided by Valrhona SA, Tain l’Hermitage, France.

### 2.3 Sample preparation and extraction procedure

Black and brown cocoa beans were prepared and their interest compounds were extracted in triplicate based on the procedure described in our previous work (25). Briefly, each cocoa bean sample (50 g) was ground under liquid nitrogen using a Pulverisette 2 analytical laboratory grinder from Fritsch (Idar-Oberstein, Germany), and stored at −80°C. An aliquot (0.12 g) of each ground sample was defatted with hexane (3 mL) by sonication (90 min, 40°C), followed by centrifugation (3,000 rpm, 10 min.), elimination of the supernatant, and drying of the defatted residue under vacuum using a Genevac evaporator (60 min, 35°C). Then, 0.015 g of defatted cocoa powder was first sonicated (5 min, 28°C) with 75 μL of methanol/acetic acid (98/2, v/v), followed by another period of sonication (30 min, 28°C.) with an additional volume (900 μL) of acetone/water/acetic acid (70/28/2, v/v/v). After centrifugation (15,000 rpm, 15 min), 700 μL of supernatant were dried under vacuum (35°C). The dried extracts were stored at −20°C until analysis.

### 2.4 Color analysis

The color analysis for each sample was performed on an extract solution after solubilization and dilution of the dried extract obtained in the previous section. The dried extract was suspended with 700 μL of methanol/water (80/20, v/v), vortexed, sonicated (15 min), and centrifuged (15,000 rpm, 15 min). The supernatant was collected in a chromatographic vial and used for two purposes: chromatographic analysis, described in the next section, and color analysis. For the color analysis, 50 μL of the supernatant was diluted 20 times with 950 μL of an acidic solution prepared by mixing a 6 M HCl solution with a methanol/water solution (50/50, v/v), following a ratio of 10/940, v/v. This diluted extract was analyzed using a Shimadzu UV-1800 spectrophotometer (Canby, OR, United States), with plastic cuvettes having an optical thickness of 10 mm. Absorbance was measured every 5 nm between 380 and 700 nm. The absorbance was multiplied by a correction factor of 20, corresponding to the dilution of the solubilized extract. The color parameters corresponding to lightness (L* = 0 black to L* = 100 white), a* (a* < 0 green to a* > 0 red), and b* (b* < 0 blue to b* > 0 yellow) were calculated from the corrected absorbance values, following the method of the International Organization of Vine and Wine (Method OIV-MA-AS2-11) (26). The Adobe Photoshop® software was used to create an image of the color of the solubilized extracts from the L*, a*, and b* values (27).

### 2.5 UHPLC−Q−Orbitrap MS analyses

The black and brown cocoa bean extracts that were obtained in triplicate and solubilized with methanol/water (80/20, v/v), as previously described, were analyzed using the same Ultra-high performance liquid chromatography–quadrupole-Orbitrap high-resolution mass spectrometry (UHPLC−Q−Orbitrap MS) system, and the same UHPLC parameters, as previously reported (23). Concerning the HRMS settings, the electrospray ionization source was operated in the negative mode. The spray voltage was maintained at 2500 V. Settings of sheath, auxiliary and sweep gases were configured to 40, 10, and 2 (arbitrary units), respectively. Ion transfer tube and vaporizer temperatures were set to 280°C and 300°C, respectively. The mass range was from *m/z* 150 to *m/z* 1,500.

The samples were injected in two series of analyses. To generate a dataset for the untargeted metabolomics workflow, the first series (S1) was performed in the full scan mode with a resolution set to 240 k, except for one injection consisting of a quality control sample that was analyzed with a resolution set to 480 k for identification purposes. To generate a dataset for FBMN analysis, the second series (S2) was carried out using a data dependent mode integrating both HRMS and HRMS/MS experiments that was configured to perform HRMS/MS experiments on the five most intense ions found in the HRMS experiment. The resolution for the acquisition of HRMS and HRMS/MS spectra were set to 60 k and 30 k, respectively. The precursor ions were fragmented against nitrogen gas in a higher-energy collisional dissociation cell using a normalized collision energy (NCE) set to 30%. The sample list containing blank, quality control, and cocoa samples was organized in the same way that in a previous work (23).

Some samples were analyzed in the positive mode for the identification of peptides using a targeted method. In this case, the main settings that were modified in relation to the negative mode analysis concerned the spray voltage (3,500 V), the *m/z* range (130–900), and the NCE set to 20%.

### 2.6 Chemometrics

The HRMS data from the S1 series were processed by the 3.2 Compound Discover software using the parameters and a workflow described in the Supplementary material (text document). The resulting dataset contained the features (couples of retention time and *m/z* values) in the rows and their abundances for each sample in the columns.

3.2 Compound Discoverer was then used to perform a principal component analysis (PCA) on the dataset with the center and scale options and normalized areas. Afterwards a fold change analysis and an ANOVA were also performed on the dataset to construct a volcano plot. Explanation about the construction and interpretation of a volcano plot were previously reported (23). The thresholds of -log10 *p*-value and of log2 fold change were set to 3.3 (corresponding to a *p*-value of 0.0005) and 0.1, respectively, for the selection of the most discriminant features.

Finally, a FBMN analysis was performed for annotation purposes on the HRMS and HRMS/MS data obtained from the series of analysis S2. The data files in .raw format were converted to .mzXML format, using the MSConvert tool (version 3). Data were then processed with MZmine 3 (version 3.9.0) (28) using processing parameters described in the Supplementary material (text document). The resulting feature quantification table (.CSV file format) containing the features and their abundances, and the HRMS/MS spectral summary (.MGF file) with a representative HRMS/MS spectrum per feature were exported to GNPS (29) for FBMN analysis (24). The molecular networks were then created from the following parameters: precursor ion mass tolerance = 0.0075 Da, HRMS/MS fragment ion tolerance = 0.0075 Da, cosine score > 0.7 and number of matched peaks >6. The molecular networks were visualized using Cytoscape software (version 3.10.1) (30). Manual annotation of features was performed based on predicted molecular formulae, fragmentation patterns and comparison to standards or literature.

## 3 Results and discussion

### 3.1 Color analysis

In general, the colors of the black and brown cocoa bean extracts injected into the UHPLC system were different from each other. The color analysis on these extracts revealed that these sample groups were mostly different in terms of the a* color parameter (Table 1). The mean values of the a* color parameter for black cocoa bean extracts ranged from 16 to 24, while for brown cocoa bean extracts the ranged from 7 to 12. These results show that the red color has a greater influence to the extract color for black bean extracts in relation to the brown bean extract. The L* mean values for most of the black cocoa bean extracts were slightly inferior to the mean values for brown cocoa bean extracts (Table 1), indicating that in general these extracts were slightly darker as compared to the brown cocoa bean extracts. The range of values for the parameter b* was of the same order for both the black and brown sample groups. The reconstituted color images of these extracts from their L*, a* and b* values (Table 1) were well correlated with the color perceived from the extracts.

**Table 1:** L*, a* and b* color parameters for extracts of defatted cocoa beans.

Black beans^a^	F178	F181	F182	F206	F214	F215	F216	F217
Color parameters^b^								
L*	72.3 ± 3.7	69.7 ± 0.3	69.9 ± 4.7	65.4 ± 6.2	61.3 ± 6.7	64.5 ± 4.7	65.6 ± 2.6	68.4 ± 2.4
a*	16.0 ± 2.7	18.6 ± 0.8	17.4 ± 0.6	21.4 ± 3.2	24.0 ± 1.9	20.5 ± 1.8	17.8 ± 0.9	16.0 ± 1.8
b*	30.9 ± 3.5	36.6 ± 1.2	35.3 ± 0.2	33.1 ± 3.4	35.5 ± 2.6	38.0 ± 2.2	39.2 ± 1.5	37.2 ± 2.0
Color images^c^	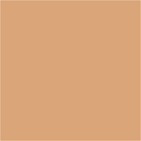	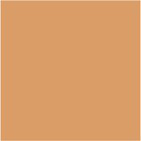	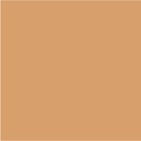	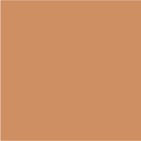	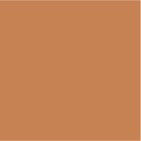	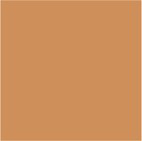	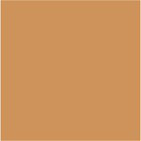	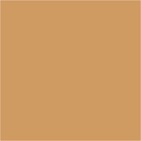
Brown beans^a^	F15	F50	F51	F54	F57	F184	F185	F186
Color parameters^b^								
L*	73.2 ± 4.3	77.5 ± 3.4	69.3 ± 5.3	75.9 ± 1.5	71.9 ± 2.5	76.8 ± 3.0	75.9 ± 7.4	69.9 ± 2.6
a*	11.4 ± 1.0	11.1 ± 1.5	11.7 ± 2.3	11.1 ± 2.7	9.7 ± 1.3	6.6 ± 0.8	7.8 ± 2.7	9.7 ± 0.6
b*	32.4 ± 1.7	29.2 ± 3.9	36.4 ± 3.8	32.3 ± 6.7	29.8 ± 3.2	33.6 ± 1.0	30.1 ± 4.7	33.4 ± 2.0
Color images^c^	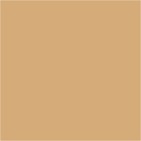	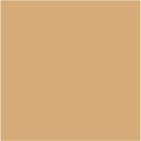	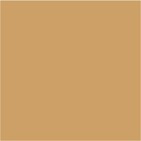	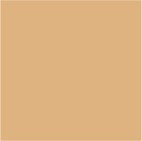	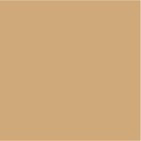	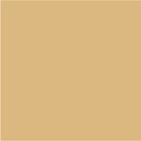	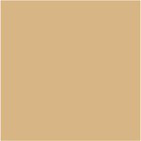	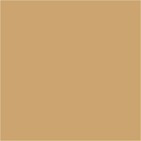

a Black and brown beans were named by analogy with the black and brown 70% cocoa dark chocolates produced from them in a previous work (23).

b The L*, a*, and b* values correspond to the mean ± standard deviation from triplicate analyses.

c Color images of black and brown cocoa bean extracts were obtained from their L*, a*, and b* color parameters by Adobe PhotoShop®.

The color difference between the two groups of extracts suggests that their respective beans also differ in terms of color. This result provides insights on the origin of the black and brown colors of 70% dark chocolates (23). These color difference preexists in the commercial beans and changes differently during processing, resulting in the final colors observed in their respective chocolates. The color difference between the bean classes reinforces the hypothesis that they also have distinct chemical profiles, which can already be inferred from the different chemical profiles of their respective chocolates (23), since they were produced using the same process from commercial beans. Finally, the different L*, a*, and b* profiles between the two groups of bean samples, especially for the values of the a* parameter, also indicate that they may be useful for quality control and selection of beans.

### 3.2 Data processing and statistical analysis

**Figure 1:**
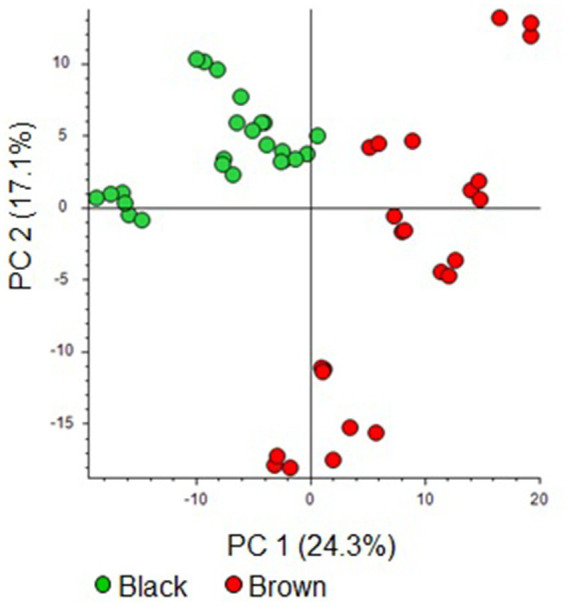
Score plot of principal component analysis from the UHPLC−HRMS features of the black and brown beans.

The processing of the cocoa bean UHPLC−HRMS data from the series of analysis S1 resulted in a dataset of 414 features (Supplementary Table S1, .xlsx file). The PCA score plot from the dataset indicates a notable separation between the brown and black cocoa bean classes, as showed in Figure 1. Principal Components 1 and 2 (PC1 and PC2) explain 24.3 and 17.1% of the dataset’s variability, respectively. This differentiation is more influenced by PC1 in comparison to PC2. Typically, black cocoa beans exhibit lower and higher scores on PC1 and PC2, respectively. On the other hand brown chocolates showed higher scores on PC1 and scattered scores along PC2.

**Figure 2:**
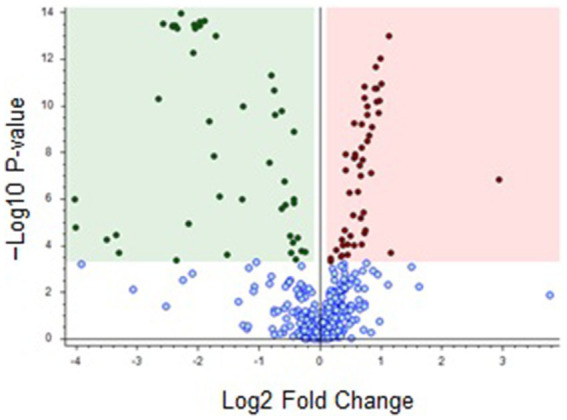
Volcano plot comparing brown versus black cocoa beans. Red (right hand side) and green (left hand side) dots represent overaccumulated discriminating features for brown and black beans, respectively, and blue dots (bottom) indicate metabolites that were not highly discriminating for either of the beans (*p*-value: < 0.0005; Log2 fold change: 0.1). The discriminating features are listed in Supplementary Table S2.

Regarding the volcano plot (Figure 2) derived from the dataset (Supplementary Table S1), the overaccumulated discriminating compounds for brown and black cocoa beans were situated on the right and left hand side of the graph, respectively.

As described in Figure 2, the features above the threshold of -log10 *p*-value = 3.3 on the volcano plot corresponded to highly discriminant features. Therefore, the green shaded region on the left of Figure 2 contains the most discriminating features for black cocoa beans (45 metabolites coded from FK1 to FK45 in Supplementary Table S2) and the red shaded region on the right contains the most discriminating features for brown beans (50 metabolites coded as FN46-FN95). The metabolites are listed in Supplementary Table S2 in descending order of significance, according to Figure 2.

The discriminating features for black and brown cocoa beans were emphasized on the loading plot (Supplementary Figure S1) of the previously described PCA (Figure 1). Supplementary Figure S1 shows that these features played a significant role in the separation between black and brown cocoa beans, as depicted in the PCA score plot (Figure 1).

### 3.3 Compound identification

The UHPLC method of the present work was the same that was used in our previous work on dark chocolates (23). Additionally, the HRMS/MS method using the negative mode was also very similar, the main difference corresponds to the fragmentation mode. In the present work, a NCE of 30% was used instead of a stepped NCE of 20–40–60% (23). This last fragmentation energy was too strong for some compounds, leading to additional fragmentation of intermediate ions. Although better HRMS/MS spectra were obtained with a NCE at 30% for most compounds, compared with the stepped NCE (data not shown), the characteristic fragment ions of the compounds could be detected in both modes. Consequently, the annotation process of the cocoa bean features was based on our previous UHPLC-HRMS/MS data (23) and supported by molecular networking analysis.

#### 3.3.1 Identification of discriminating compounds for black cocoa beans

Sixteen of forty-five discriminating features for black cocoa beans were annotated as phenolic compounds. Thirteen features showed similar chromatographic (retention times) and mass spectral information (proposed molecular formula and HRMS/MS fragmentation patterns; Supplementary Table S2) than the corresponding features found in black chocolates (23). Therefore they were annotated as protocatechuic acid (FK3), (epi)catechin-*O*-pentosides (FK2 and FK6), (epi)catechin-*O*-hexosides (FK4 and FK5), (epi)gallocatechin-*O*-hexosides (FK9 and FK11), oxidized A-type procyanidin dimer (FK1), A-type procyanidin dimer *O*-pentosides (FK10 and FK13), A-type procyanidin dimer *O*-hexoside (FK7), A-type procyanidin trimer pentoside (FK12) and A-type procyanidin trimer hexoside (FK8). Furthermore, some of these compounds such as protocatechuic acid (FK3) (31), (epi)catechin-*O*-hexoside, A-type procyanidin dimer *O*-hexoside (10, 32), A-type procyanidin trimer hexoside, A-type procyanidin dimer *O*-pentoside and A-type procyanidin trimer *O*-pentoside (19, 32) were already reported for cocoa beans.

**Figure 3:**
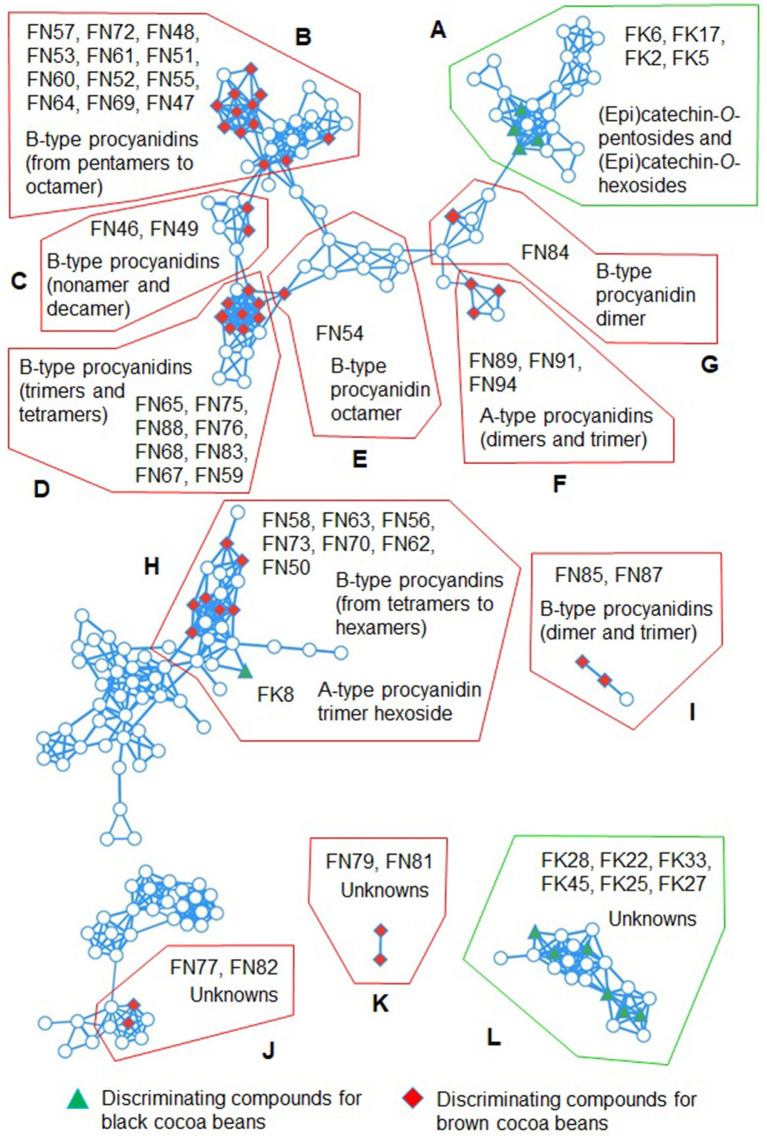
Feature-based molecular networks with clusters **(A–L)** containing discriminating compounds for black and brown cocoa beans. Full analytical details on the compounds are available in Supplementary Table S2. FK and FN indicate discriminating compounds for black and brown cocoa beans, respectively.

The FK17, FK20 and FK42 did not correspond to any feature in our previous work (23). The first feature had the same predicted molecular formula and similar HRMS/MS fragmentation pattern than the features FK4 and FK5. Additionally, it was present in a cluster (Cluster A in Figure 3) of the molecular networks along with other flavanol glycosides, indicating their HRMS/MS spectral similarities. Therefore, it was annotated as (epi)catechin-*O*-hexoside. FK20 had the same predicted molecular formula than FK1, indicating that these two compounds are isomers. However, their HRMS/MS fragments are quite different. Indeed, oxidized A-type procyanidin dimers can have different isomers and different MS/MS fragmentation patterns (33). Oxidized A-type procyanidins are degradation compounds of A-type procyanidins and they are characterized by a third ether-linkage between the flavanol units that can be located at different positions (13, 33). FK20 feature had some fragments characteristics of flavanols, the fragment ion at *m/z* 447.0708 was formed due to the neutral loss of 126 Da, which can corresponds to the loss of the phloroglucinol A-ring (33). This moiety also generated a fragment ion at *m/z* 125.0243. The fragment ion at *m/z* 435.0717 can also corresponds to the loss of another A-ring (neutral loss of 138 Da) (33), that was also observed as a fragment ion at *m/z* 137.0244. Likely, this compound is also an oxidized A-type procyanidin dimer, but its structure could not be hypothesized based on the obtained fragments. FK1 and FK20 were probably formed during fermentation and/or drying processes. To the best of our knowledge, this is the first time that this type of compound was evidenced in cocoa beans. The molecular formula and fragmentation pattern of FK42 corresponded to quercetin-3-*O*-glucoside which was confirmed by comparison with standard and literature (10).

Three features (FK29 − FK31; Supplementary Table S2) were suspected to be tripeptides. The molecular ion at *m/z* 391.1984 for FK29 corresponded to the molecular formula C_19_H_28_O_5_N_4_ (error = −0.83 ppm). This ion presented fragments (*m/z* 276.1719 and *m/z* 129.1033) whose mass difference (147.0685 Da) corresponded to phenylalanine (C_9_H_9_ON, 0.86 ppm). The comparison between the molecular formulas of FK29 and FK30, as well as between FK30 and FK31, corresponded to a difference of one oxygen atom and C_2_H_4_ (2 x CH_2_), respectively, suggesting that small chemical differences distinguish these compounds.

The targeted analysis of these compounds in the positive mode in a quality control sample, as well in some cocoa bean samples showed more informative fragmentation spectra, which allowed for the proposal of their amino acid sequences by *de novo* sequencing based on Roepstorff and Fohlman (34) and Johnson and colleagues (35) nomenclature. The mass difference between the molecular ion at *m/z* 393.2138 and the fragment at *m/z* 280.1291 (y2 ion) in the HRMS/MS spectrum of FK29 (Supplementary Table S3; Supplementary Figure S2) corresponded to (iso)leucine loss on the N-terminus (133.0847 Da, C_6_H_11_ON, 5.54 ppm). Similarly, the mass difference between the fragments *m/z* 280.1291 (y2 ion) and *m/z* 133.0608 (y1 ion) corresponded to phenylalanine loss (147.0683 Da, C_9_H_9_ON, −0.91 ppm). The presence of asparagine could be deduced from the remaining mass needed to complete the molecular ion mass. The b2 ion (Leu/Ile-Phe) was detected at *m/z* 261.1598. The fragmentation pattern of this tripeptide (Supplementary Figure S2) corresponded to the sequence Leu/Ile-Phe-Asn (Supplementary Table S3).

HRMS/MS spectrum of FK30 showed a molecular ion at *m/z* 409.2083 and a fragment ion at *m/z* 310.1399 (y2 ion, Supplementary Table S3; Supplementary Figure S3). The difference between these ions corresponded to valine (99.0684 Da, C_5_H_9_ON, 0.16 ppm). The difference between this y2 ion and the y1 ion at *m/z* 182.0812 corresponded to glutamine (128.0587 Da, C_5_H_8_O_2_N_2_, 0.88 ppm). Tyrosine was assigned as the remaining amino acid to complete the molecular formula. The b2 and b2-17 ions (Val-Gln) were detected at *m/z* 228.1343 and 211.1077, respectively. Thus, based on the fragmentation pattern (Supplementary Figure S3), the sequence of this tripeptide was proposed as Val-Gln-Tyr (Supplementary Table S3). A tripeptide VQY has already been described in fermented and dried cocoa beans (7).

Similarly, the fragmentation of the molecular ion of FK31 at *m/z* 381.1776 (Supplementary Table S3; Supplementary Figure S4) resulted in a loss of serine (*m/z* 381.1776 – *m/z* 294.1448 y2 ion = 87.0329 Da, C_3_H_5_O_2_N, 9.55 ppm), glutamine (*m/z* 294.1448 y2 ion – *m/z* 166.0862 y1 ion = 128.0585 Da, C_5_H_8_O_2_N_2_, −0.53 ppm). Phenylalanine was assigned as the remaining amino acid to complete the molecular formula. The b2 and b2-17 ions (Ser-Gln) were detected at *m/z* 216.0978 and 199.0714, respectively. The fragmentation pattern of this compound (Supplementary Figure S4) corresponded to Ser-Gln-Phe (Supplementary Table S3). A tripeptide with the same molecular formula, but without information about the sequence or composition of amino acids, was already reported in fermented and dried cocoa beans (21).

Several discriminating features for black cocoa beans remained unkwnon (Supplementary Table S2). Some of them (FK22, FK25, FK27, FK28, FK33 and FK45) had similar fragmentation patterns and were observed in a same cluster (Cluster L in Figure 3) in the molecular networks, suggesting that they belong to the same compound family.

#### 3.3.2 Identification of discriminating compounds for brown cocoa beans

The majority of the discriminating compounds for brown cocoa beans were phenolic compounds (41 compounds). Among them, 27 compounds were also reported in our previous work (23) and corresponded to (epi)catechin hexoside-sulfate (FN90), dehydrodicatechin B with *β*-interflavanic configuration/B-type procyanidin dimer (FN92), A-type procyanidin dimer (FN94), B-type procyanidin trimers (FN59, FN68, FN76, FN83, and FN85), B-type procyanidin tetramers (FN62, FN65, FN67, FN70, FN75, and FN78), B-type procyanidin pentamers (FN50, FN51, FN53, FN60, FN64, and FN73), B-type procyanidin hexamers (FN56, FN58, FN61, FN63), B-type procyanidin heptamer (FN52), B-type procyanidin octamer (FN47), and B-type procyanidin nonamer (FN49). FN92 likely corresponds to two co-eluted isomers because its HRMS/MS spectrum shows specific fragment of dehydrodicatechins B at *m/z* 393.0979 and B-type procyanidins at *m/z* 451.1027 (36). This last fragment was not detected in brown cocoa beans in our previous work, where this feature corresponded only to a dehydrodicatechin B, coded as N45 (23). Dias and colleagues (37) also noted the co-elution of these types of isomers and employed ion mobility spectrometry to separate the compounds. All the aforementioned procyanidins have been observed in cocoa beans (19), and dehydrodicatechins B in roasted beans (14).

A-type procyanidin dimer (FN89), B-type procyanidin trimer (FN88), B-type procyanidin pentamer (FN74), B-type procyanidin hexamers (FN48 and FN72), B-type procyanidin heptamers (FN55, FN57, and FN69), and B-type procyanidin octamer (FN54) were annotated by comparing their HRMS/MS spectral data with their respective isomers described above.

FN84 and FN87 were annotated as procyanidin B2 and procyanidin B5, respectively, based on their HRMS/MS fragmentation patterns (36) and comparison with standard compounds. FN91 corresponded to an A-type procyanidin trimer due to its HRMS/MS fragmentation pattern that was similar to those reported in the literature (19). Additionally, this compound was connected to other A-type procyanidins in the molecular networks (Cluster F). The doubly charged ion of FN46 at *m/z* 1440.3173 corresponded to the formula of a B-type procyanidin decamer (C_150_H_122_O_60_, −0.13 ppm). In addition, its HRMS/MS fragments were also similar to those observed for a triply charged decamer (19). All these compounds have previously been detected in cocoa beans (19).

FN93 at *m/z* 849.2036 (C_45_H_38_O_17_, −0.01 ppm) could not be identified. Nevertheless, it exhibited HRMS/MS fragments at *m/z* 289.0718, 245.0818, 165.0193, 137.0244, and 125.0244, which are indicative of (epi)catechin structure (14). Lin and colleagues (38) detected B-type proanthocyanidin trimers having the same molecular formula than FN93 in mangosteen, but their fragmentation patterns are quite different from that found in the present study, although they also observed the fragment corresponding to (epi)catechin at *m/z* 289. Consequently, FN93 was annotated as an (epi)catechin derivative. The majority of the phenolic compounds in brown cocoa beans are represented in the molecular networks within clusters A − I in Figure 3.

An amino acid derivative was annotated as feruloyl aspartic acid (FN80). It was previously observed in black cocoa beans (23). The pairs of compounds FN77 and FN82, as well as FN79 and FN81, were connected in the molecular networks, with each pair assigned to a different cluster (Figure 3, Clusters J and K, respectively). This suggests that each pair corresponds to similar structures, but they could not be annotated. Compounds FN66, FN71, FN86, and FN95 also remained unidentified.

### 3.4 Comparison between black and brown cocoa beans

Among the 16 discriminanting phenolic compounds of black cocoa beans, 14 belong to the flavanol family. FK3 is a hydroxybenzoic acid, and FK42 is a flavonol. These flavanols have a low degree of polymerization ranging from monomers to trimers, and most of them are glycosylated compounds (FK2, FK4, FK5, FK6, FK7, FK8, FK9, FK10, FK11, FK12, FK13, and FK17). In addition, the oligomers belong to the subclass of A-type procyanidins (FK1, FK7, FK8, FK10, FK12, FK13, and FK20).

Concerning the brown cocoa beans, all 41 discriminanting phenolic compounds are also flavanols. Unlike what was observed in black cocoa beans, most of these compounds have a higher degree of polymerization and are not glycosylated (39 compounds ranging from dimers to decamers). Moreover, they are predominantly B-type procyanidins (36 compounds), while only three compounds are A-type procyanidin trimer and dimers (FN91, FN94 and FN89).

Concerning the amino acid derivatives, three discriminanting tripeptides (FK29, FK30, and FK31) were annotated in black cocoa beans and one discriminanting N-phenylpropenoyl-L-amino acid (FN80) was annotated in brown beans.

The annotated discriminating compounds may be potential markers of black and brown cocoa beans, and may be useful for quality control purposes in order to improve the quality of their corresponding chocolates.

In relation to the beans and their respective chocolates (23), black cocoa beans and black chocolates had similar profiles in terms of discriminating phenolic compounds. They have 13 phenolics in common as described in the 3.3.1 section. FK17, FK20 and FK42 were not reported in chocolates. Conversely, an A-type procyanindin dimer *O*-hexoside and an A-type procyanidin dimer *O*-pentoside, previously observed in black chocolates and coded as K4 and K9 (23), were not discriminating compounds for black cocoa beans.

Concerning brown beans and brown chocolates (23), both also showed similar phenolic profiles in terms of compound families, but several differences were observed in terms of the individually observed compounds. Of the 41 discriminating phenolic compounds annotated in the beans, 27 were also discriminating compounds for the chocolates. FN46, FN48, FN54, FN55, FN57, FN69, FN72, FN74, FN84, FN87, FN88, FN89, FN91, and FN93 were exclusive discriminant compounds for the beans. On the other hand, the chocolates presented 9 phenolic compounds that were exclusively discriminant for them, comprising A-type and B-type procyanidins, B-type procyanidin *C*-glycosides, a dehydrodicatechin B, a phenolic acid and a (epi)catechin derivative (coded as N42, N44, N49, N53, N54, N55, N65, N71, and N75 in the work of Dias and colleagues (23).

During cocoa processing, the amount of phenolic compounds decreases and they undergo different chemical transformations (5, 17). Flavanols, in particular, can undergo oxidation (14, 15), epimerization (12, 13), polymerization and depolymerization during this processing (16, 17). The differences observed in terms of discriminating phenolic compounds between the beans of the present work and their respective chocolates (23) implies that the ratio of the brown/black bean abundances for these compounds were altered during the cocoa processing. The black and brown beans in the present study were processed in the same manner for the production of chocolates that were studied by Dias and colleagues (23). It is not possible to precisely explain why several compounds mentioned above were discriminants for black or brown beans but not for their respective chocolates based on our data. Since the chemical compositions of black and brown beans differ, at least at the level of polyphenols, the ratio of the brown/black bean abundances for these compounds was likely altered during processing due to different chemical interactions of these compounds in each type of beans. Taeye and colleagues (14) studied the synergistic effect between different flavanols in model solutions at 60°C. They showed that epicatechin strongly accelerated the degradation of a B-type procyanidin dimer, leading to the formation of a trimer.

For the compounds that were discriminant only for the chocolates (23), there might have been a synthesis process for some of these compounds during cocoa processing that contributed to them becoming discriminants. A-type procyanidin dimer *O*-hexoside and an A-type procyanidin dimer *O*-pentoside (K4 and K9) (23), which were discriminating compounds for black chocolates, as mentioned earlier, maybe had an increase in their abundances during processing due to their formation from the epimerization of their isomers, which were discriminating compounds for black beans (FK7, FK13, and FK10). Epimerization of flavanols was observed under cocoa bean roasting conditions (13, 14).

B-type procyanidins, described above as discriminating compounds for brown chocolates (23), but not for brown beans, have a low degree of polymerization. This suggests that these compounds may have had their abundances increased during processing due to the depolymerization of precursors that are abundant in brown beans (B-type procyanidins having a higher degree of polymerization). This depolymerization may have been followed by oxidation to form other compounds that were also discriminant only for brown chocolates (A-type procyanidin dimer and dehydrodicatechin B, described above), since B-type procyanidins may be oxidized into A-type procyanidins (14) and (epi)catechins may be oxidized to form dehydrodicatechins B (39). Alternatively, these oligomers may also have been formed by epimerization from their isomers present in brown beans.

Stark and Hofmann (40) demonstrated that flavanols can be *C*-glycosylated in the presence of glucose, under alkaline conditions and at high temperatures (80°C). *C*-glycosilation could be a possibility that may have contributed to the *C*-glycosylated B-type procyanidins described above becoming discriminant compounds for brown chocolates.

Taken together, these results show that the majority of the discriminating phenolic compounds for black and brown chocolates (23) were already pre-existing and discriminant in their respective cocoa beans. However, as compared to black beans, brown beans exhibited a more diverse phenolic profile, containing several compounds that were not discriminant for their chocolates. Finally, these results also provide insights into possible origins of compounds that were discriminant for the chocolates, although they cannot be confirmed in the present study. In order to increase the knowledge on the characteristic phenolic profile of black chocolates and black cocoa beans, further studies on the influence of drying, fermentation and genetic origin on the phenolic profile of cocoa beans may be conducted.

Considering the unknown compounds (Supplementary Table S2), black beans had many more compounds (26 unknown compounds) than brown beans (8 compounds), reinforcing the differences in chemical profiles between these beans. Comparing beans to chocolates, black cocoa beans exhibited a greater number of unknown compounds compared to black chocolates (23). Only the compounds FK22, FK15, FK25, FK27, FK14, and FK23 (coded as K16, K18, K19, K22, K24, and K25, respectively) (23) were also discriminating compounds for black chocolates. On the other hand, the differences were less pronounced between brown beans and brown chocolates. They shared five unknown compounds in common (FK79, FK81, FK95, FK86 and FK82 that were coded as N35, N41, N57, N69 and N73, respectively, in our previous work) (23). These results indicate a high variability between black chocolates and their beans in terms of unknown discriminating compounds. Moreover, these data show that cocoa processing, including roasting and conching, had a strong impact mainly on the unknown compounds of black cocoa beans. To gain a better understanding of the overall profile of discriminating compounds in both cocoa beans and black chocolates, further studies are needed to identify these unknown compounds in both types of samples.

Although this work focused on discriminant compounds, regardless whether they are or not responsible for the cocoa bean colors, the markers found may indirectly contribute to the observed colors. The discriminant phenolic compounds (monomeric flavanols and procyanidins) are colorless, but they are precursors of colored compounds formed through enzymatic and/or non-enzymatic oxidation during the fermentation and drying of cocoa beans. Concerning the discriminant compounds derived from amino acids, they can participate in Maillard reactions, which generate colored products during the drying of the beans (23).

## 4 Conclusion

This study aimed to evaluate the color and the discriminating compounds for two types of cocoa beans, referred to as black and brown beans, which were used to produce black and brown chocolates (70% cocoa content) in a previous work (23). The present work showed that the color difference between those chocolates preexists in their respective beans. Black and brown beans had 45 and 50 discriminating compounds, respectively. The majority of them were annotated as phenolic compounds: 16 for black and 41 for brown beans. The majority of the phenolics for black beans were *O*-glycosylated flavanols with a low degree of polymerization (from monomers to trimers), of which the oligomers were A-type procyanidins. In contrast, the compounds for brown beans were mainly non-glycosylated B-type procyanidins having a higher degree of polymerization (from dimers to decamers). Considering the discriminant amino acid derivatives, three tripeptides and one N-phenylpropenoyl-L-amino acid were annotated for black and brown beans, respectively. These discriminating compounds may be useful as markers for quality control purposes and may contribute to the selection of beans that yield dark chocolates having brown or black colors.

A comparison with the literature on black and brown chocolates (23) produced from black and brown beans of the present work, respectively, revealed that, especially for the brown samples, several phenolics were discriminating compounds solely for the beans, while others were exclusive to the chocolates. This suggests chemical transformations such as depolymerization, oxidation, epimerization, and *C*-glycosylation during the processing of cocoa beans, which may be confirmed in future works. Additionally, most discriminating phenolic compounds for black and brown chocolates were also discriminant for their respective beans. Further metabolomic studies could investigate the influence of drying, fermentation, and genetic origin on the chemical profiles of these cocoa beans.

## Data Availability

The datasets presented in this article are not readily available because of a confidentiality agreement with the company Valrhona. Requests to access the datasets should be directed to the corresponding author.
