# Supplementary material for: Cocoa bean metabolomics reveals polyphenols as potential markers relating to fine dark chocolate color shades
Source: Front Nutr. 2024 Sep 25;11:1467282. doi: 10.3389/fnut.2024.1467282 (PMC11463152; doi:10.3389/fnut.2024.1467282)
Supplement: Supplementary file 2 [file Data_Sheet_1.docx]

Supplementary Material

# Description of the Compound Discoverer software processing parameters used for raw UHPLC−HRMS data processing and selection of features

*Select Spectra*

Spectrum Properties Filter:

- Lower RT Limit: 0.9

- Upper RT Limit: 17

- Lowest Charge State: 0

- Highest Charge State: 3

- Min. Precursor Mass: 150 Da

- Max. Precursor Mass: 1500 Da

- Total Intensity Threshold: 0

- Minimum Peak Count: 1

Peak Filters:

- S/N Threshold (FT-only): 3

General Settings:

- Precursor Selection: Use MS(n - 1) Precursor

- Use Isotope Pattern in Precursor Reevaluation: True

*Align Retention Times*

General Settings:

- Alignment Model: Adaptive curve

- Alignment Fallback: None

- Maximum Shift [min]: 1

- Mass Tolerance: 2 ppm

- Remove Outlier: True

*Detect Compounds*

General Settings:

- Mass Tolerance [ppm]: 1 ppm

- Intensity Tolerance [%]: 50

- S/N Threshold: 2

- Min. Peak Intensity: 2000000

- Ions: [2M-H]-1; [M+Cl]-1; [M+Formic Acid-H]-1; [M-2H]-2; [M-2H+Na]-1, [M-H]-1

- Base Ions: [M-H]-1

- Min. Element Counts: C H O

- Max. Element Counts: C190 H190 N8 O120 S2

Peak Detection:

- Filter Peaks: False

- Max. Peak Width [min]: 0.6

- Remove Singlets: True

- Min. # Scans per Peak: 5

- Min. # Isotopes: 2

Isotope Grouping:

- Min. Spectral Distance Score: 0

- Remove Potentially False Positive Isotopes: True

*Group Compounds*

Compound Consolidation:

- Mass Tolerance: 2 ppm

- RT Tolerance [min]: 0.6

*Fill Gaps*

General Settings:

- Mass Tolerance: 2 ppm

- S/N Threshold: 2

*Apply Missing Value Imputation*

General Settings:

- Imputation Method: Median + Small Value with Variability

*Apply QC Correction*

General Settings:

- Regression Model: Linear

- Min. QC Coverage [%]: 40

- Max. QC Area RSD [%]: 30

- Max. Corrected QC Area RSD [%]: 25

- Max. # Files Between QC Files: 15

*Normalize Areas*

General Settings:

- Normalization Type: Constant Median

- Exclude Blanks: True

*Mark Background Compounds*

General Settings:

- Max. Sample/Blank: 5

- Max. Blank/Sample: 0

- Hide Background: True

# Description of the MZmine software processing parameters used for raw UHPLC−HRMS/MS data and feature selection before feature-based molecular networking analysis

*Mass detection*

Scans: MS level (1)

Scan types: All scan types

Mass detector: Centroid

- Noise level: 1000000.0

- Detect isotope signals below noise level: false

- *m/z* tolerance: 5.0E-4 *m/z* or 10.0 ppm

- Maximum charge of isotope *m/z*: 1

Scans: MS level (2)

Scan types: All scan types

Mass detector: Centroid

- Noise level: 1000.0

- Detect isotope signals below noise level: false

- *m/z* tolerance: 5.0E-4 *m/z* or 10.0 ppm

- Maximum charge of isotope *m/z*: 1

*ADAP Chromatogram Builder*

Scans: MS level (1)

Group intensity threshold: 1000000.0

Min highest intensity: 1000000.0

Scan to scan accuracy (*m/z*): 0.002 *m/z* or 5.0 ppm

*Local minimum feature resolver*

MS/MS scan pairing: true

- Retention time tolerance: 0.2 minutes

- MS1 to MS2 precursor tolerance (*m/z*): 0.002 *m/z* or 5.0 ppm

Dimension: Retention time

Chromatographic threshold: 0.9

Minimum relative height: 0.0

Minimum absolute height: 1000000.0

Min ratio of peak top/edge: 1.7

Peak duration range (min/mobility): [0.02..1.0]

Min # of data points: 5

*13C isotope filter (formerly: isotope grouper)*

*m/z* tolerance: 0.001 *m/z* or 3.0 ppm

Retention time tolerance: 0.01 minutes

Maximum charge: 2

Representative isotope: Most intense

Never remove feature with MS2: true

*Join aligner*

*m/z* tolerance: 0.0 *m/z* or 5.0 ppm

Weight for *m/z*: 75.0

Retention time tolerance: 0.05 minutes

Weight for RT: 25.0

Require same charge state: false

*Filtering feature list rows*

Minimum features in a row (abs or %): true (2.0)

Minimum features in an isotope pattern: true (2)

Validate 13C isotope pattern: true

- *m/z* tolerance: 0.0005 *m/z* or 5.0 ppm

- Max charge: 2

- Estimate minimum carbon: true

- Remove if 13C: true

Feature with MS2 scan: true

Never remove feature with MS2: true

*Feature list blank subtraction*

Minimum # of detection in blanks: 3

Fold change increase: true (3.0)

*Gap filling*

Intensity tolerance: 0.1

*m/z* tolerance: 0.002 *m/z* or 5.0 ppm

Retention time tolerance: 0.05 minutes

Minimum data points: 1

*Duplicate feature list rows filter*

Filter mode: NEW AVERAGE

*m/z* tolerance: 0.0 *m/z* or 5.0 ppm

RT tolerance: 0.07 minutes

# Supplementary Figures and Tables

## Supplementary Figures


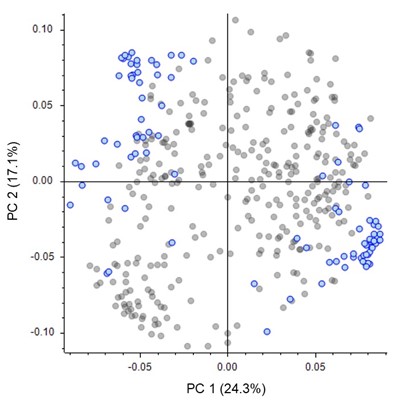


**Supplementary Figure S1.** Loading plot of principal component analysis from the UHPLC−HRMS features of the black and brown cocoa beans. Blue dots correspond to discriminating features for black (left group) and brown (right group) beans obtained from the differential analysis in Figures 2. The corresponding score plot of PCA is shown in Figure 1.

**
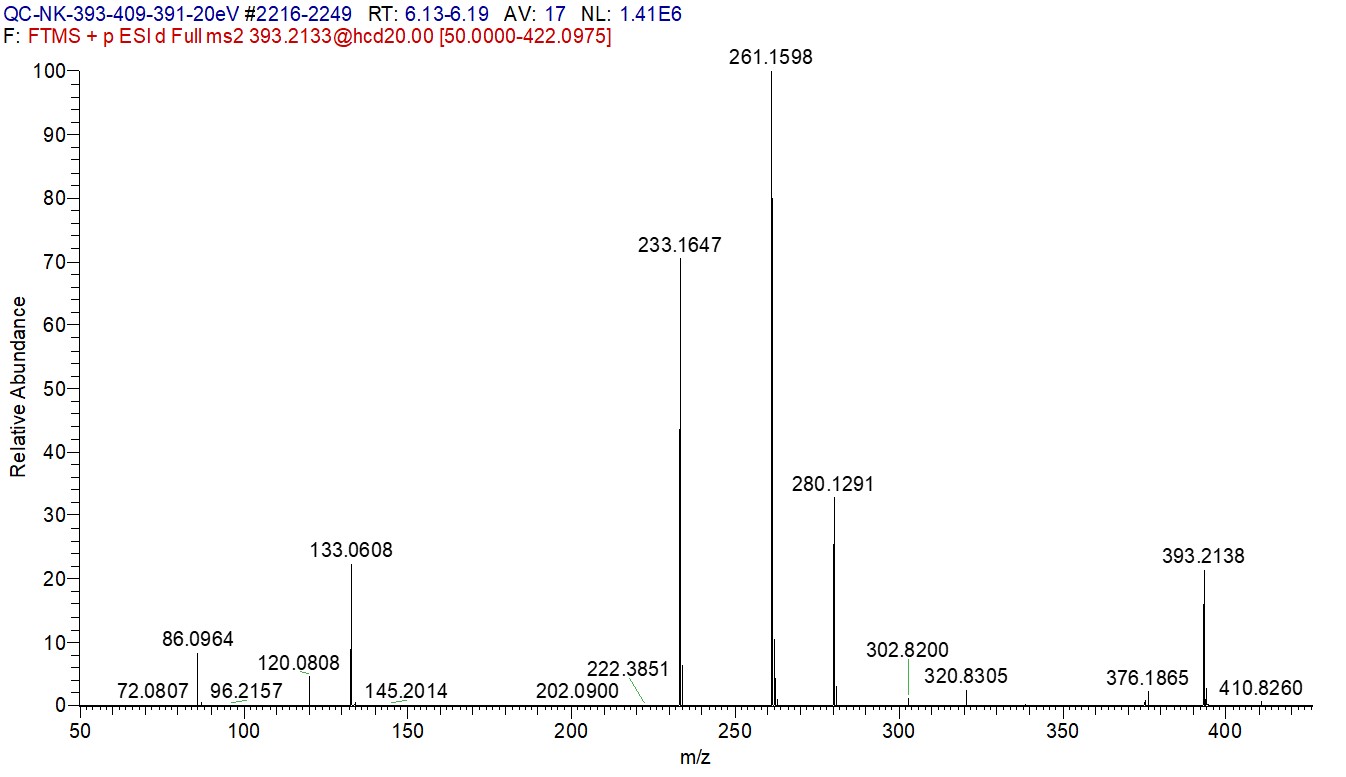
**

**Supplementary Figure S2.** Positive mode HRMS/MS spectrum from the molecular ion at *m/z* 393.2135 corresponding to a tripeptide (I)LFN (compound FK29 in Table S3).

**
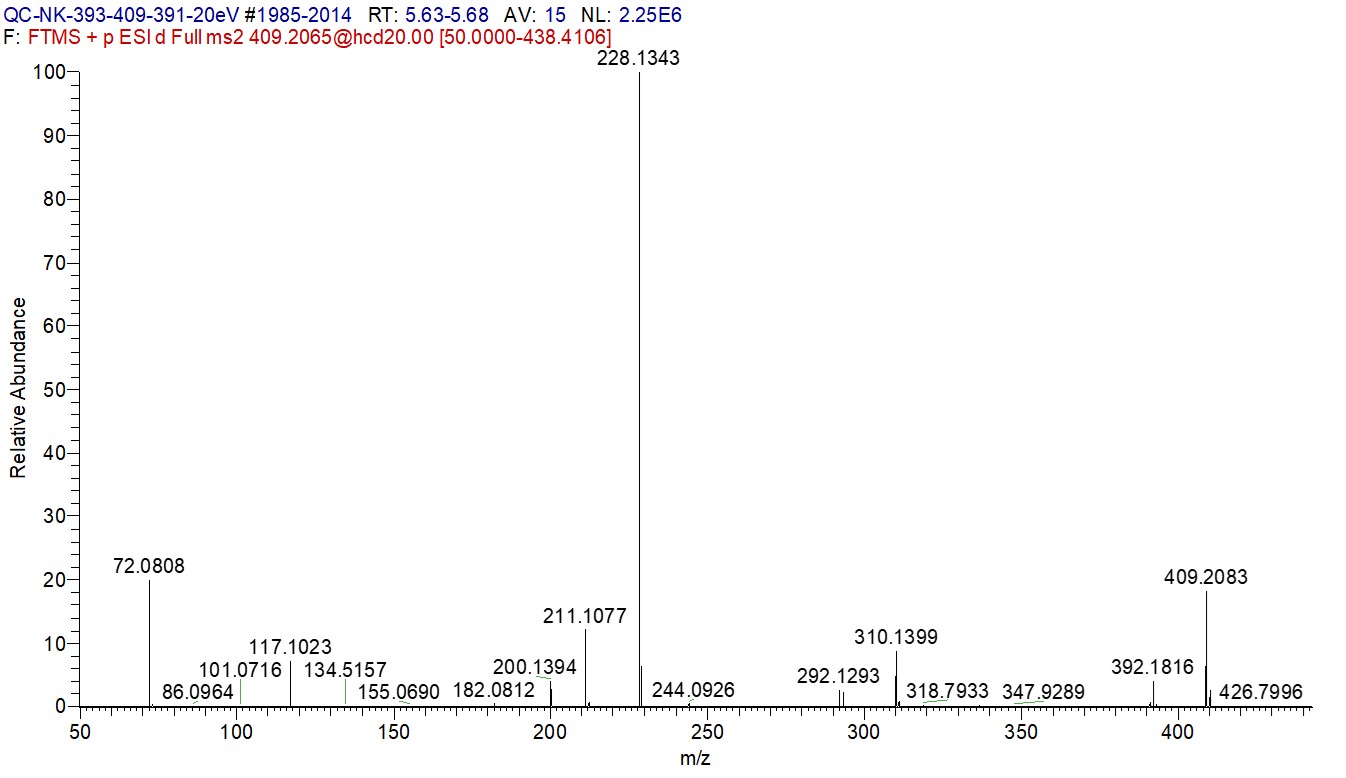
**

**Supplementary Figure S3.** Positive mode HRMS/MS spectrum from the molecular ion at *m/z* 409.2082 corresponding to a tripeptide VQY (compound FK30 in Table S3).

**
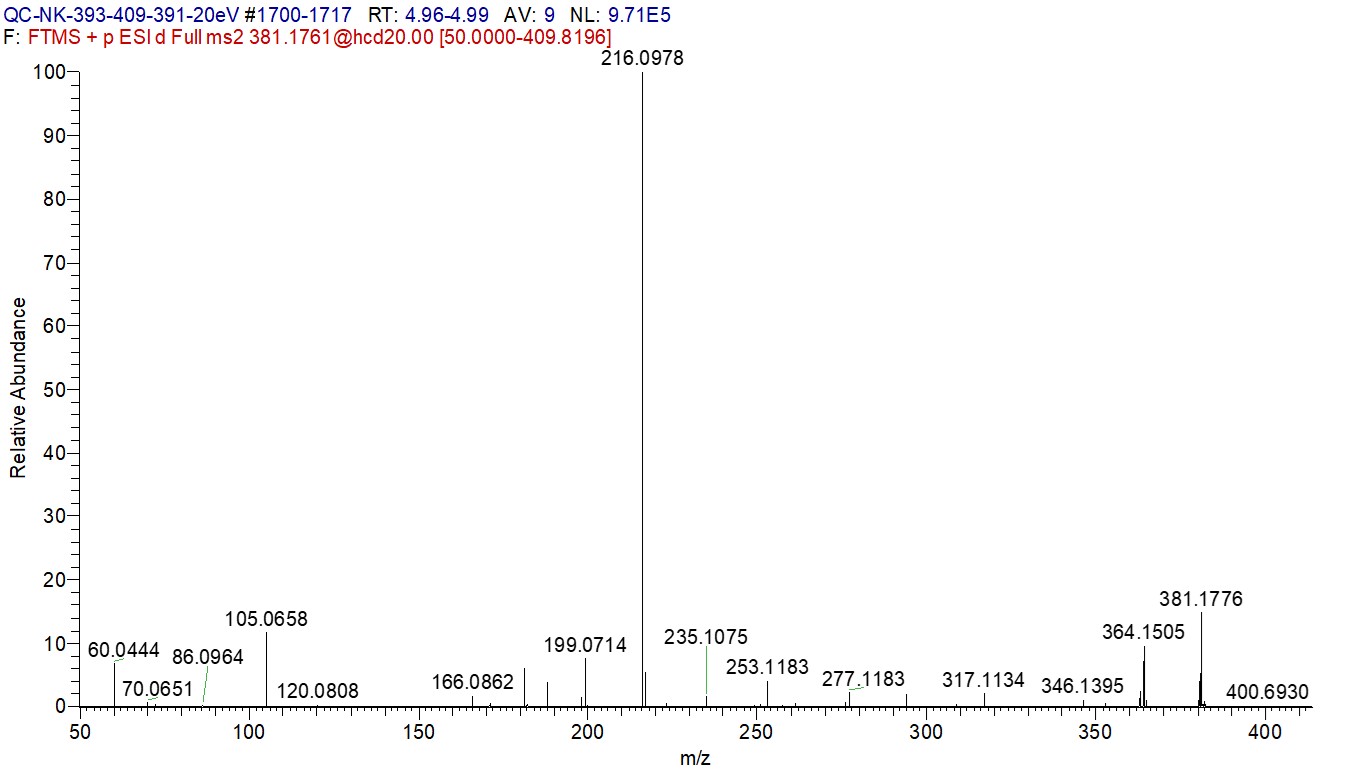
**

**Supplementary Figure S4.** Positive mode HRMS/MS spectrum from the molecular ion at *m/z* 381.1771 corresponding to a tripeptide SQF (compound FK31 in Table S3).

## Supplementary Tables

**Supplementary Table S2.** Highly discriminating compounds for black and brown cocoa beans tentatively annotated by UHPLC−ESI−Q−Orbitrap MS analyses.

| **Comp. Code^a^** | **Rt (min)** | **Ion type** | **Experimental *m/z*** | **Calculated molecular weight (Da)** | **Formula (M)** | **Error (ppm)** | **MS/MS ion fragments (relative abundance in %)** | **Annotation** | **Ref.** |  |
| --- | --- | --- | --- | --- | --- | --- | --- | --- | --- | --- |
| FK1 | 13.94 | [M ̶ H] ^̶^ | 573.1039 | 574.1112 | C_30_H_22_O_12_ | 0.06 | 529.1132 (8), 447.0714 (59), 429.0609 (10), 339.0508 (8), 325.0353 (22), 285.0404 (100) | Oxidized A-type procyanidin dimer | (33,13,23) | |
| FK2 | 6.82 | [M ̶ H] ^̶^ | 421.1136 | 422.1209 | C_20_H_22_O_10_ | -0.93 | 289.0717 (100), 245.0820 (11), 146.9385 (11) | (Epi)catechin-*O*-pentoside | (23) | |
| FK3 | 1.93 | [M ̶ H] ^̶^ | 153.0193 | 154.0266 | C_7_H_6_O_4_ | -0.19 | 153.0193 (65), 109.0295 (100) | Protocatechuic acid^b^ | (23) | |
| FK4 | 6.17 | [M ̶ H] ^̶^ | 451.1242 | 452.1315 | C_21_H_24_O_11_ | -0.92 | 289.0719 (100), 245.0818 (12) | (Epi)catechin-*O*-hexoside | (23) | |
| FK5 | 5.08 | [M ̶ H] ^̶^ | 451.1242 | 452.1315 | C_21_H_24_O_11_ | -0.87 | 405.0436 (12), 289.0711 (100) | (Epi)catechin-*O*-hexoside | (23) | |
| FK6 | 7.74 | [M ̶ H] ^̶^ | 421.1137 | 422.1209 | C_20_H_22_O_10_ | -0.89 | 421.1139 (17), 289.0718 (100), 245.0820 (23) | (Epi)catechin-*O*-pentoside | (23) | |
| FK7 | 12.70 | [M ̶ H] ^̶^ | 737.1724 | 738.1796 | C_36_H_34_O_17_ | 0.03 | 611.1404 (36), 539.0982 (68), 449.087 (100), 448.0793 (45), 407.0765 (10), 289.0718 (16), 287.0561 (17) | A-type procyanidin dimer *O*-hexoside | (23) | |
| FK8 | 11.15 | [M ̶ H] ^̶^ | 1025.2354 | 1026.2427 | C_51_H_46_O_23_ | -0.28 | 855.1783 (15), 735.1569 (18), 657.1040 (20), 609.1250 (31), 573.1249 (45), 537.0826 (29), 451.1031(10), 447.0938 (75), 429.0613 (18), 393.0615 (44), 375.0507 (13), 349.0716 (28), 307.0613 (59), 289.0719 (100), 245.0818 (14), 243.0300 (41), 137.0243 (11) | A-type procyanidin trimer hexoside | (23) | |
| FK9 | 5.64 | [M ̶ H] ^̶^ | 467.1191 | 468.1263 | C_21_H_24_O_12_ | -0.95 | 467.1194 (13), 449.1085 (12), 305.0668 (72), 287.0562 (100), 269.0458 (13), 195.0299 (38) 161.0244 (60), 125.0244 (84) | (Epi)gallocatechin-*O*-hexoside | (23) | |

**Supplementary Table S2.** Continued.

| **Comp. Code^a^** | **Rt (min)** | **Ion type** | **Experimental *m/z*** | **Calculated molecular weight (Da)** | **Formula (M)** | **Error (ppm)** | **MS/MS ion fragments (relative abundance in %)** | **Annotation** | **Ref.** |
| --- | --- | --- | --- | --- | --- | --- | --- | --- | --- |
| FK10 | 8.13 | [M ̶ H] ^̶^ | 707.1616 | 708.1689 | C_35_H_32_O_16_ | -0.19 | 581.1302 (23), 539.0976 (61), 449.0870 (100), 448.0791 (43), 407.0761 (11), 287.0562 (14) | A-type procyanidin dimer *O*-pentoside | (23) |
| FK11 | 4.61 | [M ̶ H] ^̶^ | 467.1191 | 468.1264 | C_21_H_24_O_12_ | -0.76 | 467.1195 (15), 449.1087 (12), 305.0666 (75), 287.0562 (100), 269.0455 (14), 195.0299 (40), 161.0245 (60), 125.0243 (80) | (Epi)gallocatechin-*O*-hexoside | (23) |
| FK12 | 12.14 | [M ̶ H] ^̶^ | 995.2250 | 996.2322 | C_50_H_44_O_22_ | -0.23 | 825.1672 (16), 705.1464 (20), 691.1289 (15), 673.1192 (12), 657.1040 (18), 579.1143 (17), 543.1145 (64), 537.0827 (27), 429.0611 (16), 417.0826 (37), 393.0613 (46), 349.0717 (27), 307.0613 (58), 289.0718 (100), 245.0821 (13), 243.0299 (36), 137.0244 (11) | A-type procyanidin trimer pentoside | (23) |
| FK13 | 13.02 | [M ̶ H] ^̶^ | 707.1615 | 708.1689 | C_35_H_32_O_16_ | -0.19 | 581.1298 (24), 539.0980 (64), 449.0870 (100), 448.0792 (37), 407.0766 (9), 289.0717 (9), 287.0561 (15) | A-type procyanidin dimer *O*-pentoside | (23) |
| FK14 | 6.87 | [M ̶ H] ^̶^ | 414.2356 | 415.2428 | C_17_H_37_O_10_N | 2.63 | 414.2354 (100), 370.2462 (16), 242.1875 (8), 144.0778 (53), 74.0247 (17) | Unknown | (23) |
| FK15 | 9.78 | [M ̶ H] ^̶^ | 860.4119 | 861.4190 | C_34_H_59_O_15_N_11_ | -0.20 | 860.4112 (68), 842.4009 (14), 830.4001 (100), 812.3909 (21), 800.3896 (67), 486.2312 (13) | Unknown | (23) |
| FK16 | 15.80 | [M ̶ H] ^̶^ | 419.1920 | 420.1992 | C_19_H_32_O_10_ | -0.78 | 125.0244 (19), 119.035 (14), 113.0243 (10), 101.0244 (46), 99.0451 (95), 89.0243 (30), 73.0294 (11), 71.0138 (11), 59.0138 (59), 57.0345 (100) | Unknown |  |
| FK17 | 5.79 | [M ̶ H] ^̶^ | 451.1242 | 452.1315 | C_21_H_24_O_11_ | -0.86 | 451.1253 (37), 289.0725 (100), 245.0825 (28) | (Epi)catechin-*O*-hexoside | (23) |
| FK18 | 4.78 | [M ̶ H] ^̶^ | 632.2896 | 633.2968 | C_25_H_43_O_12_N_7_ | -0.22 | 632.2895 (18), 614.2786 (31), 588.2638 (22), 570.2528 (54), 473.2363 (12), 328.1149 (14), 310.1044 (100), 292.0934 (12), 259.1409 (28), 229.1305 (64), 213.0878 (16), 195.0775 (31), 114.0198 (10) | Unknown |  |

**Supplementary Table S2.** Continued.

| **Comp. Code^a^** | **Rt (min)** | **Ion type** | **Experimental *m/z*** | **Calculated molecular weight (Da)** | **Formula (M)** | **Error (ppm)** | **MS/MS ion fragments (relative abundance in %)** | **Annotation** | **Ref.** |
| --- | --- | --- | --- | --- | --- | --- | --- | --- | --- |
| FK19 | 9.24 | [M ̶ H] ^̶^ | 931.4492 | 932.4564 | C_37_H_64_O_16_N_12_ | 0.08 | 931.4492 (55), 913.4383 (11), 901.4376 (92), 883.4267 (20), 871.4270 (58), 557.2690 (10), 201.0710 (100) | Unknown |  |
| FK20 | 8.11 | [M ̶ H] ^̶^ | 573.1037 | 574.1110 | C_30_H_22_O_12_ | -0.23 | 573.1034 (100), 529.1139 (36), 487.1031 (14), 485.1237 (19), 447.0708 (9), 445.0920 (10), 437.0874 (51), 435.0717 (59), 403.0820 (12), 393.0975 (17), 391.0822 (33), 349.0717 (13), 311.0562 (52), 299.0561 (71), 271.0612 (40), 137.0244 (40), 125.0243 (12) | Oxidized A-type procyanidin dimer | (33,13) |
| FK21 | 4.77 | [M ̶ H] ^̶^ | 397.2201 | 398.2273 | C_17_H_30_O_5_N_6_ | -1.08 | 397.2203 (100), 355.1985 (36), 353.2304 (16), 336.2043 (10), 311.2091 (17), 223.1089 (30), 173.1043 (96), 131.0825 (31) | Unknown |  |
| FK22 | 10.04 | [M ̶ H] ^̶^ | 391.1606 | 392.1679 | C_17_H_28_O_10_ | -0.95 | 247.1188 (66), 161.0454 (29), 125.0244 (17), 101.0244 (30), 99.0452 (95), 59.0138 (63), 57.0345 (100) | Unknown | (23) |
| FK23 | 6.51 | [M ̶ H] ^̶^ | 688.3536 | 689.3609 | C_30_H_47_O_8_N_11_ | -0.08 | 644.3267 (100), 627.3008 (17), 602.3057 (18), 184.0729 (17) | Unknown | (23) |
| FK24 | 5.48 | [M ̶ H] ^̶^ | 482.3093 | 483.3165 | C_22_H_41_O_5_N_7_ | -0.85 | 482.3090 (100), 440.2874 (21), 173.1043 (32), 131.0826 (10) | Unknown |  |
| FK25 | 5.18 | [M ̶ H] ^̶^ | 425.1661 | 426.1733 | C_17_H_30_O_12_ | -0.95 | 281.1154 (100), 263.1054 (22), 245.1029 (26), 119.0715 (61), 101.0237 (60), 99.0462 (35), 59.0141 (42), 57.0344 (62) | Unknown | (23) |
| FK26 | 1.82 | [M ̶ H] ^̶^ | 483.2569 | 484.2642 | C_21_H_36_O_7_N_6_ | -0.81 | 483.2572 (100), 209.0931 (19), 145.0982 (17), 128.0353 (10), 127.0513 (50) | Unknown |  |
| FK27 | 5.33 | [M ̶ H] ^̶^ | 425.1661 | 426.1734 | C_17_H_30_O_12_ | -0.83 | 281.1243 (100), 263.1137 (23), 161.0457 (15), 125.0244 (13), 101.0245 (16), 99.0452 (36), 59.0138 (19), 57.0345 (29) | Unknown | (23) |
| FK28 | 10.91 | [M ̶ H] ^̶^ | 437.2023 | 438.2096 | C_19_H_34_O_11_ | -1.10 | 293.1608 (86), 161.0454 (19), 125.0244 (23), 101.0244 (35), 99.0451 (100), 59.0138 (80), 57.0345 (93) | Unknown |  |

**Supplementary Table S2.** Continued.

| **Comp. Code^a^** | **Rt (min)** | **Ion type** | **Experimental *m/z*** | **Calculated molecular weight (Da)** | **Formula (M)** | **Error (ppm)** | **MS/MS ion fragments (relative abundance in %)** | **Annotation** | **Ref.** |
| --- | --- | --- | --- | --- | --- | --- | --- | --- | --- |
| FK29 | 6.38 | [M ̶ H] ^̶^ | 391.1984 | 392.2056 | C_19_H_28_O_5_N_4_ | -0.83 | 391.1981 (57), 276.1719 (90), 217.0982 (21), 146.0611 (60), 131.0463 (56), 129.1033 (54), 114.0196 (100), 113.0357 (10), 96.0091 (41) | L/IFN tripeptide^c^ |  |
| FK30 | 5.84 | [M ̶ H] ^̶^ | 407.1933 | 408.2005 | C_19_H_28_O_6_N_4_ | -0.89 | 407.1930 (64), 363.2033 (23), 292.1667 (21), 226.1197 (49), 180.0667 (100), 172.1092 (17), 127.0513 (12) | VQY tripeptide^c^ |  |
| FK31 | 5.12 | [M ̶ H] ^̶^ | 379.1620 | 380.1693 | C_17_H_24_O_6_N_4_ | -0.84 | 379.1614 (100), 305.1622 (51), 234.1241 (21), 231.1118 (11), 227.8676 (11), 214.0848 (26), 213.1022 (11), 201.0707 (79), 199.0977 (18), 184.0733 (71), 164.0723 (80), 128.0359 (13), 127.0516 (33) | SQF tripeptide^c^ |  |
| FK32 | 8.20 | [M ̶ H] ^̶^ | 202.1085 | 203.1157 | C_9_H_17_O_4_N | -0.08 | 202.1084 (100), 158.1185 (24), 130.0871 (15) | Unknown |  |
| FK33 | 11.20 | [M ̶ H] ^̶^ | 437.2023 | 438.2096 | C_19_H_34_O_11_ | -1.15 | 293.1607 (85), 161.0454 (26), 125.0244 (23), 101.0244 (35), 99.0451 (100), 59.0138 (73), 57.0345 (96) | Unknown |  |
| FK34 | 12.53 | [M ̶ H] ^̶^ | 357.1222 | 358.1294 | C_13_H_26_O_9_S | -0.90 | 357.1222 (100), 241.0025 (1), 96.9600 (3) | Unknown |  |
| FK35 | 9.67 | [M ̶ H] ^̶^ | 782.3688 | 783.3761 | C_33_H_53_O_13_N_9_ | -0.93 | 782.3682 (19), 738.3423 (100), 721.3155 (14), 607.2480 (15), 552.2783 (16), 550.2265 (12), 453.2100 (62), 398.2036 (10), 284.1253 (22), 267.0989 (13) | Unknown |  |
| FK36 | 5.92 | [M ̶ H] ^̶^ | 467.2143 | 468.2216 | C_21_H_32_O_8_N_4_ | -0.83 | 423,1881 (100), 393,1780 (23), 349,1879 (21), 180,0666 (35) | Unknown |  |
| FK37 | 10.45 | [M ̶ H] ^̶^ | 435.1868 | 436.1940 | C_19_H_32_O_11_ | -1.05 | 291.1447 (66), 161.0453 (25), 125.0245 (21), 101.0244 (32), 99.0452 (100), 59.0138 (89), 57.0345 (93) | Unknown |  |
| FK38 | 11.56 | [M ̶ H] ^̶^ | 619.2731 | 620.2805 | C_28_H_40_O_10_N_6_ | -0.22 | 361.1398 (11), 343.1295 (100), 299.1399 (12), 227.1150 (43) | Unknown |  |
| FK39 | 7.25 | [M ̶ H] ^̶^ | 719.3007 | 720.3080 | C_31_H_44_O_12_N_8_ | 0.14 | 719.2996 (39), 701.2890 (11), 689.2887 (100), 347.1360 (35), 341.1462 (28) | Unknown |  |

**Supplementary Table S2.** Continued.

| **Comp. Code^a^** | **Rt (min)** | **Ion type** | **Experimental *m/z*** | **Calculated molecular weight (Da)** | **Formula (M)** | **Error (ppm)** | **MS/MS ion fragments (relative abundance in %)** | **Annotation** | **Ref.** |
| --- | --- | --- | --- | --- | --- | --- | --- | --- | --- |
| FK40 | 6.53 | [M ̶ H] ^̶^ | 485.3088 | 486.3161 | C_22_H_42_O_6_N_6_ | -0.96 | 485.3091 (100), 215.1512 (22), 130.0873 (15) | Unknown |  |
| FK41 | 4.95 | [M ̶ H] ^̶^ | 373.1170 | 374.1243 | C_18_H_14_O_2_N_8_ | 0.80 | 373.1172 (100), 241.0025 (1), 96.9601 (3) | Unknown |  |
| FK42 | 12.53 | [M ̶ H] ^̶^ | 463.0879 | 464.0951 | C_21_H_20_O_12_ | -0.77 | 463.0880 (27), 301.0353 (58), 300.0277 (100), 201.0709 (20) | Quercetin-3-*O*-glucoside^b^ | (10) |
| FK43 | 13.69 | [M ̶ H] ^̶^ | 489.1973 | 490.2047 | C_22_H_34_O_12_ | -0.67 | 183.1025 (100), 139.1128 (20), 125.0244 (21), 101.0244 (20), 99.0451 (44), 57.0345 (42) | Unknown |  |
| FK44 | 15.41 | [M ̶ H] ^̶^ | 745.3890 | 746.3963 | C_35_H_54_O_10_N_8_ | 0.01 | 701.3629 (100), 684.3353 (12), 473.2763 (15), 341.1575 (18) | Unknown |  |
| FK45 | 2.67 | [M ̶ H] ^̶^ | 411.1504 | 412.1576 | C_16_H_28_O_12_ | -1.06 | 267.1086 (100), 249.0981 (24), 161.0457 (23), 125.0245 (21), 101.0244 (22), 99.0452 (55), 59.0138 (30), 57.0346 (51) | Unknown |  |
| FN46 | 12.67 | [M ̶ 2H]^2 ̶^ | 1440.3173 | 2882.6492 | C_150_H_122_O_60_ | -0.13 | 575.1190 (29), 449.0871 (33), 423.0712 (20), 407.0765 (31), 405.0613 (31), 289.0719 (23), 287.0560 (100), 245.0455 (22), 243.0298 (29) | B-type procyanidin decamer | (19) |
| FN47 | 12.12 | [M ̶ 2H]^2 ̶^ | 1152.2539 | 2306.5219 | C_120_H_98_O_48_ | -0.37 | 737.1532 (11), 693.1260 (10), 575.1185 (27), 451.1038 (12), 449.0876 (37), 423.0717 (22), 407.0756 (43), 289.0712 (38), 287.0562 (100), 161.0243 (39) | B-type procyanidin octamer | (23) |
| FN48 | 11.02 | [M ̶ 2H]^2 ̶^ | 864.1912 | 1730.3965 | C_90_H_74_O_36_ | 0.32 | 575.1192 (15), 451.1033 (12), 449.0868 (25), 407.0764 (52), 289.0716 (77), 287.0560 (100), 161.0244 (44) | B-type procyanidin hexamer | (23) |
| FN49 | 12.44 | [M ̶ 2H]^2 ̶^ | 1296.2853 | 2594.5851 | C_135_H_110_O_54_ | -0.40 | 575.1187 (24), 449.0870 (30), 407.0764 (32), 289.0718 (29), 287.0560 (100) | B-type procyanidin nonamer | (23) |
| FN50 | 9.29 | [M ̶ 2H]^2 ̶^ | 720.1589 | 1442.3325 | C_75_H_62_O_30_ | -0.08 | 577.1359 (6), 575.1199 (8), 449.0880 (19), 407.0772 (52), 289.0716 (100), 151.0400 (22) | B-type procyanidin pentamer | (23) |

**Supplementary Table S2.** Continued.

| **Comp. Code^a^** | **Rt (min)** | **Ion type** | **Experimental *m/z*** | **Calculated molecular weight (Da)** | **Formula (M)** | **Error (ppm)** | **MS/MS ion fragments (relative abundance in %)** | **Annotation** | **Ref.** |
| --- | --- | --- | --- | --- | --- | --- | --- | --- | --- |
| FN51 | 11.51 | [M ̶ 2H]^2 ̶^ | 720.1590 | 1442.3325 | C_75_H_62_O_30_ | -0.06 | 575.1193 (9), 449.0875 (23), 407.0768 (68), 289.0715 (100), 151.0400 (28) | B-type procyanidin pentamer | (23) |
| FN52 | 11.83 | [M ̶ 2H]^2 ̶^ | 1008.2229 | 2018.4591 | C_105_H_86_O_42_ | -0.14 | 577.1364 (15), 575.1203 (21), 451.1042 (13), 449.0854 (24), 407.0773 (50), 289.0715 (62), 287.0564 (100), 161.0246 (34) | B-type procyanidin heptamer | (23) |
| FN53 | 7.98 | [M ̶ 2H]^2 ̶^ | 720.1589 | 1442.3326 | C_75_H_62_O_30_ | -0.02 | 575.1198 (7), 451.1047 (9), 449.0872 (21),407.0772 (51), 289.0716 (100) | B-type procyanidin pentamer | (23) |
| FN54 | 12.91 | [M ̶ 2H]^2 ̶^ | 1152.2539 | 2306.5219 | C_120_H_98_O_48_ | -0.37 | 449.0878 (13), 407.0759 (22), 289.0714 (24), 287.0560 (48), 161.0243 (28), 125.0244 (100) | B-type procyanidin octamer | (23) |
| FN55 | 12.34 | [M ̶ 2H]^2 ̶^ | 1008.2229 | 2018.4604 | C_105_H_86_O_42_ | 0.49 | 577.1361 (9), 575.1201 (21), 451.1032 (12), 449.0874 (33), 425.0878 (12), 407.0760 (55), 289.0718 (57), 287.0560 (100), 161.0246 (40) | B-type procyanidin heptamer | (23) |
| FN56 | 11.27 | [M ̶ 2H]^2 ̶^ | 864.1912 | 1730.3960 | C_90_H_74_O_36_ | 0.04 | 577.1344 (9), 575.1210 (15), 451.1028 (13), 449.0870 (26), 407.0768 (66), 289.0715 (100), 287.0559 (94), 161.0243 (47) | B-type procyanidin hexamer | (23) |
| FN57 | 12.61 | [M ̶ 2H]^2 ̶^ | 1008.2222 | 2018.4594 | C_105_H_86_O_42_ | 0.03 | 577.1356 (9), 575.1195 (24), 451.1030 (14), 449.0880 (34), 425.0883 (11), 407.0769 (63), 289.0718 (83), 287.0558 (100), 161.0243 (43) | B-type procyanidin heptamer | (23) |
| FN58 | 11.58 | [M ̶ 2H]^2 ̶^ | 864.1912 | 1730.3960 | C_90_H_74_O_36_ | 0.02 | 577.1343 (8), 575.1182 (16), 451.1024 (14), 449.0865 (29), 407.0764 (63), 289.0715 (100), 287.0559 (99), 161.0244 (49) | B-type procyanidin hexamer | (23) |
| FN59 | 7.11 | [M ̶ H] ^̶^ | 865.1981 | 866.2054 | C_45_H_38_O_18_ | -0.45 | 451.1029 (13), 425.0877 (14), 407.0770 (82), 289.0717 (72), 287.0560 (56), 261.0404 (20), 245.0456 (20), 243.0299 (28), 161.0244 (28), 125.0243 (100) | B-type procyanidin trimer | (23) |

**Supplementary Table S2.** Continued.

| **Comp. Code^a^** | **Rt (min)** | **Ion type** | **Experimental *m/z*** | **Calculated molecular weight (Da)** | **Formula (M)** | **Error (ppm)** | **MS/MS ion fragments (relative abundance in %)** | **Annotation** | **Ref.** |
| --- | --- | --- | --- | --- | --- | --- | --- | --- | --- |
| FN60 | 10.97 | [M ̶ 2H]^2 ̶^ | 720.1590 | 1442.3324 | C_75_H_62_O_30_ | -0.15 | 577.1340 (5), 575.1184 (6), 449.0869 (16), 407.0765 (47), 405.0610 (19), 289.0716 (100), 151.0400 (21) | B-type procyanidin pentamer | (23) |
| FN61 | 8.88 | [M ̶ 2H]^2 ̶^ | 864.1904 | 1730.3955 | C_90_H_74_O_36_ | -0.28 | 575.1181 (6), 451.1030 (13), 449.0870 (26), 407.0768 (66), 289.0716 (90), 287.0559 (100), 161.0245 (44) | B-type procyanidin hexamer | (23) |
| FN62 | 11.18 | [M ̶ H] ^̶^ | 1153.2617 | 1154.2689 | C_60_H_50_O_24_ | -0.27 | 577.1350 (7), 575.1184 (6), 451.1022 (8), 449.0875 (12), 425.0867 (10), 423.0720 (9), 413.0876 (10), 407.0768 (52), 289.0716 (37), 287.0559 (64), 125.0244 (100) | B-type procyanidin tetramer | (23) |
| FN63 | 12.23 | [M ̶ 2H]^2 ̶^ | 864.1912 | 1730.3960 | C_90_H_74_O_36_ | 0.02 | 577.1345 (9), 575.1188 (17), 451.1025 (14), 449.0871 (33), 407.0768 (64), 289.0717 (100), 287.0560 (91), 161.0244 (47) | B-type procyanidin hexamer | (23) |
| FN64 | 10.66 | [M ̶ H] ^̶^ | 1441.3253 | 1442.3324 | C_75_H_62_O_30_ | -0.13 | 575.1182 (14), 451.1039 (11), 449.0871 (25), 425.0869 (10), 407.0767 (35), 405.0613 (32), 289.0716 (33), 287.0562 (100), 161.0244 (32) | B-type procyanidin pentamer | (23) |
| FN65 | 10.27 | [M ̶ H] ^̶^ | 1153.2617 | 1154.2690 | C_60_H_50_O_24_ | -0.22 | 577.1343 (6), 575.1193 (5), 451.1025 (11), 449.0875 (13), 425.0873 (11), 423.0719 (9), 413.0874 (10), 407.0769 (46), 405.0610 (17), 289.0715 (44), 287.0559 (67), 125.0244 (100) | B-type procyanidin tetramer | (23) |
| FN66 | 1.61 | [M ̶ H] ^̶^ | 409.1360 | 410.1433 | C_17_H_22_O_8_N_4_ | -1.23 | Not fragmented | Unknown |  |
| FN67 | 7.70 | [M ̶ H] ^̶^ | 1153.2617 | 1154.2689 | C_60_H_50_O_24_ | -0.29 | 449.0868 (14), 425.0869 (10), 423.0712 (11), 413.0878 (10), 407.0769 (44), 405.0612 (20), 289.0717 (39), 287.0562 (73), 125.0245 (100) | B-type procyanidin tetramer | (23) |
| FN68 | 13.19 | [M ̶ H] ^̶^ | 865.1985 | 866.2057 | C_45_H_38_O_18_ | -0.14 | 451.1029 (12), 425.0877 (13), 407.0766 (79), 289.0718 (63), 125.0244 (100) | B-type procyanidin trimer | (23) |
| FN69 | 10.35 | [M ̶ 2H]^2 ̶^ | 1008.2220 | 2018.4585 | C_105_H_86_O_42_ | -0.42 | 575.1181 (14), 451.1031 (17), 449.0871 (29), 407.0763 (55), 289.0717 (69), 287.0562 (100), 161.0243 (53) | B-type procyanidin heptamer | (23) |

**Supplementary Table S2.** Continued.

| **Comp. Code^a^** | **Rt (min)** | **Ion type** | **Experimental *m/z*** | **Calculated molecular weight (Da)** | **Formula (M)** | **Error (ppm)** | **MS/MS ion fragments (relative abundance in %)** | **Annotation** | **Ref.** |
| --- | --- | --- | --- | --- | --- | --- | --- | --- | --- |
| FN70 | 10.52 | [M ̶ H] ^̶^ | 1153.2617 | 1154.2690 | C_60_H_50_O_24_ | -0.21 | 575.1191 (9), 451.1022 (11), 449.0870 (35), 425.0867 (11), 423.0719 (12), 413.0879 (12), 407.0767 (51), 405.0611 (30), 289.0717 (38), 287.0559 (100), 125.0244 (89) | B-type procyanidin tetramer | (23) |
| FN71 | 2.68 | [M ̶ H] ^̶^ | 366.1303 | 367.1376 | C_16_H_21_O_7_N_3_ | -1.06 | 348.1199 (29), 331.0935 (86), 301.0831 (54), 276.0202 (33), 200.9365 (28), 156.9467 (100), 113.0356 (51), 96.0091 (24) | Unknown |  |
| FN72 | 14.01 | [M ̶ 2H]^2 ̶^ | 864.1907 | 1730.3961 | C_90_H_74_O_36_ | 0.09 | 577.1335 (12), 575.1172 (12), 451.1027 (24), 449.0871 (34), 407.0767 (100), 289.0717 (76), 287.0558 (96), 161.0245 (59) | B-type procyanidin hexamer | (23) |
| FN73 | 13.75 | [M ̶ 2H]^2 ̶^ | 720.1589 | 1442.3326 | C_75_H_62_O_30_ | -0.02 | 577.1346 (11), 449.0864 (21), 407.0767 (100), 289.0716 (66), 151.0400 (31) | B-type procyanidin pentamer | (23) |
| FN74 | 11.94 | [M ̶ 2H]^2 ̶^ | 720.1590 | 1442.3326 | C_75_H_62_O_30_ | 0.01 | 577.1331 (12), 449.0891 (20), 407.0765 (90), 289.0714 (100), 151.0403 (35) | B-type procyanidin pentamer | (23) |
| FN75 | 14.18 | [M ̶ H] ^̶^ | 1153.2620 | 1154.2688 | C_60_H_50_O_24_ | -0.32 | 451.1026 (21), 449.0877 (10), 425.0869 (12), 413.0882 (10), 407.0768 (84), 405.0607 (10), 289.0716 (52), 287.0559 (48), 125.0244 (100) | B-type procyanidin tetramer | (23) |
| FN76 | 9.42 | [M ̶ H] ^̶^ | 865.1987 | 866.2058 | C_45_H_38_O_18_ | 0.01 | 525.0821 (12), 451.1032 (14), 425.0877 (15), 407.0769 (84), 405.0618 (12), 289.0718 (79), 125.0244 (100) | B-type procyanidin trimer | (23) |
| FN77 | 2.03 | [M ̶ H] ^̶^ | 433.1347 | 434.1420 | C_18_H_26_O_12_ | -0.97 | 233.0668 (42), 125.0243 (50), 89.0244 (100), 71.0138 (40), 59.0138 (31) | Unknown |  |
| FN78 | 6.80 | [M ̶ H] ^̶^ | 1153.2615 | 1154.2687 | C_60_H_50_O_24_ | -0.43 | 577.1342 (7), 575.1210 (6), 451.1022 (8), 449.0863 (14), 425.0870 (10), 423.0724 (9), 413.0872 (9), 407.0768 (46), 405.0612 (18), 289.0716 (39), 287.0559 (64), 125.0244 (100) | B-type procyanidin tetramer | (23) |
| FN79 | 5.82 | [M ̶ H] ^̶^ | 890.3381 | 891.3456 | C_34_H_53_O_19_N_9_ | -0.20 | 467.1750 (18), 449.1693 (24), 369.1769 (100), 324.0939 (19) | Unknown | (23) |

**Supplementary Table S2.** Continued.

| **Comp. Code^a^** | **Rt (min)** | **Ion type** | **Experimental *m/z*** | **Calculated molecular weight (Da)** | **Formula (M)** | **Error (ppm)** | **MS/MS ion fragments (relative abundance in %)** | **Annotation** | **Ref.** |
| --- | --- | --- | --- | --- | --- | --- | --- | --- | --- |
| FN80 | 7.73 | [M ̶ H] ^̶^ | 308.0775 | 309.0848 | C_14_H_15_NO_7_ | -0.07 | 308.0759 (42), 246.0770 (24), 193.0505 (46), 149.0607 (100), 134.0373 (15) | Feruloyl aspartic acid | (23) |
| FN81 | 6.20 | [M ̶ H] ^̶^ | 872.3279 | 873.3351 | C_34_H_51_O_18_N_9_ | -0.16 | 854.3167 (18), 621.2156 (32), 467.1779 (45), 449.1677 (15), 369.1777 (100), 324.0951 (24) | Unknown | (23) |
| FN82 | 2.27 | [M ̶ H] ^̶^ | 433.1347 | 434.1420 | C_18_H_26_O_12_ | -0.98 | 233.0667 (41), 125.0243 (38), 89.0244 (100), 71.0138 (38), 59.0138 (35) | Unknown | (23) |
| FN83 | 10.65 | [M ̶ H] ^̶^ | 865.1987 | 866.2056 | C_45_H_38_O_18_ | -0.22 | 451.1029 (11), 425.0873 (15), 407.0766 (80), 289.0718 (58), 125.0244 (100) | B-type procyanidin trimer | (23) |
| FN84 | 7.11 | [M ̶ H] ^̶^ | 577.1349 | 578.1423 | C_30_H_26_O_12_ | -0.20 | 451.1026 (5), 425.0873 (6), 407.0769 (84), 289.0716 (100), 125.0244 (60) | B-type procyanidin dimer (B2)^b^ | (36) |
| FN85 | 12.98 | [M ̶ H] ^̶^ | 865.1985 | 866.2057 | C_45_H_38_O_18_ | -0.17 | 451.1030 (16), 425.0874 (11), 407.0767 (100), 289.0718 (57), 125.0244 (37) | B-type procyanidin trimer | (23) |
| FN86 | 5.86 | [M ̶ H] ^̶^ | 501.1470 | 502.1544 | C_18_H_30_O_16_ | 1.96 | 195.0522 (100), 179.0573 (45), 161.0453 (10), 151.0625 (83), 125.0244 (14), 101.0244 (33), 99.0451 (92), 59.0138 (16), 57.0345 (94) | Unknown | (23) |
| FN87 | 12.54 | [M ̶ H] ^̶^ | 577.1350 | 578.1423 | C_30_H_26_O_12_ | -0.25 | 451.1033 (6), 425.0876 (8), 407.0769 (69), 289.0718 (100), 125.0244 (76) | B-type procyanidin dimer (B5)^b^ | (36) |
| FN88 | 6.77 | [M ̶ H] ^̶^ | 865.1981 | 866.2054 | C_45_H_38_O_18_ | -0.48 | 451.1029 (14), 425.0873 (14), 407.0768 (82), 289.0717 (83), 287.0561 (54), 261.0404 (21), 245.0456 (20), 243.0299 (30), 161.0244 (31), 125.0244 (100) | B-type procyanidin trimer | (23) |
| FN89 | 7.12 | [M ̶ H] ^̶^ | 575.1195 | 576.1267 | C_30_H_24_O_12_ | -0.18 | 539.0984 (39), 449.0871 (30), 423.0722 (43), 407.0769 (34), 287.0560 (88), 285.0405 (76), 125.0244 (100) | A-type procyanidin dimer | (23) |

**Supplementary Table S2.** Continued.

| **Comp. Code^a^** | **Rt (min)** | **Ion type** | **Experimental *m/z*** | **Calculated molecular weight (Da)** | **Formula (M)** | **Error (ppm)** | **MS/MS ion fragments (relative abundance in %)** | **Annotation** | **Ref.** |
| --- | --- | --- | --- | --- | --- | --- | --- | --- | --- |
| FN90 | 5.19 | [M ̶ H] ^̶^ | 531.0811 | 532.0884 | C_21_H_24_O_14_S | -0.48 | 531.0811 (100), 289.0717 (32), 245.0820 (2), 137.0244 (5) | (Epi)catechin hexoside-sulfate | (23) |
| FN91 | 9.43 | [M ̶ H] ^̶^ | 863.1833 | 864.1906 | C_45_H_36_O_18_ | 0.49 | 693.1241 (27), 575.1196 (29), 449.0878 (43), 411.0715 (78), 407.0775 (38), 289.0717 (93), 287.0561 (32), 285.0405 (100), 243.0297 (17), 125.0243 (62) | A-type procyanidin trimer | (19) |
| FN92 | 7.44 | [M ̶ H] ^̶^ | 577.1349 | 578.1423 | C30 H26 O12 | -0.19 | 577.1346 (100), 559.1248 (17), 533.1450 (18), 451.1027 (4), 439.1032 (75), 425.0881 (32), 393.0979 (61), 269.0454 (42), 137.0243 (27) | Dehydrodicatechin B^d^ /B-type procyanidin dimer | (23,36) |
| FN93 | 11.20 | [M ̶ H] ^̶^ | 849.2036 | 850.2109 | C45 H38 O17 | -0.01 | 435.1082 (11), 433.0926 (13), 407.0765 (48), 389.0656 (13), 289.0718 (100), 271.0612 (47), 245.0818 (13), 165.0193 (10), 137.0244 (7), 125.0244 (83) | B-type proanthocyanidin | (38) |
| FN94 | 12.52 | [M ̶ H] ^̶^ | 575.1193 | 576.1265 | C30 H24 O12 | -0.41 | 575.1187 (8), 539.0974 (39), 449.0885 (44), 423.0717 (19), 407.0775 (28), 285.0404 (100), 125.0244 (39) | A-type procyanidin dimer (A2)^b^ | (23) |
| FN95 | 9.20 | [M ̶ H] ^̶^ | 378.1667 | 379.1740 | C18 H25 N3 O6 | -1.00 | 378.1668 (3), 360.1562 (11), 343.1294 (100), 299.1399 (18), 164.0717 (22), 152.0717 (15), 147.0451 (3) | Unknown | (23) |

^a^FK and FN indicates discriminating compounds for black and brown cocoa beans, respectively, ^b^Annotation confirmed by authentic standard, ^c^The proposed amino acid sequence is based on the positive ion mode HRMS/MS data (see Table S3), ^d^Compound having a *β*-interflavanic configuration, corresponding to a biphenyl interflavanic linkage (37). Rt, retention time. The gray rows correspond to phenolic compounds that were exclusive discriminants for the beans, while the other phenolics in the non-highlighted rows were also reported as discriminants for the respective chocolates in a previous work (23).

**Supplementary Table S3.** Tripeptides annotated by UHPLC−ESI−Q−Orbitrap MS analyses in the positive ion mode.

| **Comp. Code^a^** | **Rt (min)** | **Ion type** | **Experimental *m/z*** | **Calculated molecular weight (Da)** | **Formula (M)** | **Error (ppm)** | **MS/MS ion fragments (relative abundance in %)** | **Sequence** | **Ref.** |
| --- | --- | --- | --- | --- | --- | --- | --- | --- | --- |
| FK29 | 6.16 | [M + H] ^+^ | 393.2135 | 392.2063 | C_19_H_28_O_5_N_4_ | 0.72 | 393.2138 (21), 376.1865 (2), 320.8305 (2), 280.1291 (33), 261.1598 (100), 233.1647 (69), 133.0608 (22), 120.0808 (5), 86.0964 (9) | LFN or IFN |  |
| FK30 | 5.65 | [M + H] ^+^ | 409.2082 | 408.2010 | C_19_H_28_O_6_N_4_ | -0.19 | 409.2083 (18), 392.1816 (4), 391.1979 (1), 310.1399 (9), 293.1135 (2), 292.1293 (3), 244.0926 (1), 228.1343 (100), 211.1077 (12), 200.1394 (4), 182.0812 (0.5), 117.1023 (7), 72.0808 (21) | VQY | (7) |
| FK31 | 4.98 | [M + H] ^+^ | 381.1771 | 380.1698 | C_17_H_24_O_6_N_4_ | 0.63 | 381.1776 (15), 364.1505 (9), 346.1395 (1), 317.1134 (2), 294.1448 (2), 277.1183 (2), 253.1183 (4), 235.1075 (2), 216.0978 (100), 199.0714 (7), 188.1030 (4), 181.0607 (6), 166.0862 (2), 105.0658 (12), 60.0444 (7) | SQF | (21) |

^a^FK indicates discriminating compounds for black cocoa beans. Rt, retention time.
